# The conference effect: National surgery meetings are associated with increased mortality at trauma centers without American College of Surgeons verification

**DOI:** 10.1371/journal.pone.0214020

**Published:** 2019-03-26

**Authors:** Peter C. Jenkins, Scott Painter, Teresa M. Bell, Jeffrey A. Kline, Ben L. Zarzaur

**Affiliations:** 1 Department of Surgery, Indiana University School of Medicine, Indianapolis, Indiana, United States of America; 2 Department of Surgery, University of Illinois College of Medicine in Peoria, Peoria, Illinois, United States of America; 3 Department of Emergency Medicine, Indiana University School of Medicine, Indianapolis, Indiana, United States of America; Yokohama City University, JAPAN

## Abstract

**Background:**

Thousands of physicians attend scientific conferences each year. While recent data indicate that variation in staffing during such meetings impacts survival of non-surgical patients, the association between treatment during conferences and outcomes of a surgical population remain unknown. The purpose of this study was to examine mortality resulting from traumatic injuries and the influence of hospital admission during national surgery meetings.

**Study design:**

Retrospective analysis of in-hospital mortality using data from the Trauma Quality Improvement Program (2010–2011). Identified patients admitted during four annual meetings and compared their mortality with that of patients admitted during non-conference periods. Analysis included 155 hospitals with 12,256 patients admitted on 42 conference days and 82,399 patients admitted on 270 non-conference days. Multivariate analysis performed separately for hospitals with different levels of trauma center verification by state and American College of Surgeons (ACS) criteria.

**Results:**

Patient characteristics were similar between meeting and non-meeting dates. At ACS level I and level II trauma centers during conference versus non-conference dates, adjusted mortality was not significantly different. However, adjusted mortality increased significantly for patients admitted to trauma centers that lacked ACS trauma verification during conferences versus non-conference days (OR 1.2, p = 0.008), particularly for patients with penetrating injuries, whose mortality rose from 11.6% to 15.9% (p = 0.006).

**Conclusions:**

Trauma mortality increased during surgery conferences compared to non-conference dates for patients admitted to hospitals that lacked ACS trauma level verification. The mortality difference at those hospitals was greatest for patients who presented with penetrating injuries.

## Introduction

Variation in hospital staffing has been associated with increases in risk of patient morbidity and mortality. Mortality for both critically ill patients and those requiring emergent surgical intervention, for example, has been found to increase during nights and weekends, the so-called “weekend effect.” [1, 2] Similarly, complications due to medical errors appear to increase during periods when the inexperience of medical trainees is greatest, commonly known as the “July effect.” [3–5] These fluctuations in outcomes, whether due to reduced staffing or clinical experience, reflect a challenge to hospitals’ ability to maintain healthcare quality. Hospital accreditation programs, such as those that govern trauma verification, have sought to mitigate the risk due to such fluctuations with requirements for staffing and resources. In fact, studies have credited the requirements of trauma center accreditation specifically with the absence of a weekend effect in trauma centers;[6–9] and largely as a result of its rigorous accreditation process, the U.S. trauma system has long been considered a model of care by the Institute of Medicine.[10]

Despite efforts to maintain high levels of quality healthcare delivery at all times, trauma centers continue to experience regular fluctuations in staffing, and the potential risks associated with those periods remains largely unclear. Multiple times each year, thousands of surgeons leave their respective institutions to travel to academic conferences. A recent study found that during national cardiology meetings, mortality decreased for high-risk patients hospitalized with heart failure and cardiac arrest.[11] As the authors noted, their results could be explained by a decline or absence of specialized physicians during conferences, as well as the pursuit of alternative interventions during those periods. Currently, no evidence exists regarding the influence of a “conference effect” on trauma hospital operations and the outcomes of injured patients.

In this study, we examine the association between national surgery conferences and in-hospital trauma mortality. We also perform subgroup analysis on specific trauma populations and hospitals according to their different forms of trauma center accreditation. We hypothesize that mortality resulting from traumatic injuries increases during national surgery conferences, presumably due to fluctuations in staffing.

## Methods

### Study design

We conducted a retrospective cohort study of trauma patients treated at hospitals participating in the American College of Surgeons Trauma Quality Improvement Program (ACS TQIP).[12] The primary exposure variable was hospital admission during national surgery conferences, and the control group consisted of patients admitted three weeks before and after each conference. The primary outcome of interest was in-hospital trauma mortality.

### Data source

The study population consisted of patients meeting inclusion criteria for the ACS TQIP.[12] TQIP is a consortium of trauma centers across the United States and Canada that collects clinical trauma registry data using standardized definitions and provides risk-adjusted performance improvement reports to its participants. Trained trauma registry personnel collect pre-hospital, emergency department, operative, intensive care and hospital data for all adult trauma patients with an Abbreviated Injury Scale Score of ≥ 3 in at least one body region resulting in an Injury Severity Score (ISS) ≥ 9. Regular audits ensure data validity for the program’s clinical registry. In addition to standard clinical information, the dataset for this study included date and time of hospital admission and discharge. These data were provided in an encrypted fashion through collaboration with the ACS to ensure HIPAA compliance. ACS TQIP analytic methods have been previously described in detail elsewhere.[12]

### Study population

We included injured patients admitted to TQIP participating centers between January 2010 and December 2011. The data excludes children (age less than 16 years) and patients who lacked records for date and time of admission to the Emergency Department. We excluded patients who presented to emergency departments without signs of life, defined as an initial systolic blood pressure of zero, heart rate of zero, and Glasgow Coma Scale motor score of one.[13] Rates of missing data were low (<6%) and distributed evenly between cohorts, so we excluded patients with missing data in a case-wise fashion.

### Exposure variables

The primary exposure variable was hospital admission during national surgery conferences that are consistently well attended by trauma surgeons. Conferences included annual meetings of the American College of Surgeons, the Eastern Association for the Surgery of Trauma, the Western Trauma Association, and the American Association for the Surgery of Trauma. We selected these conferences *a priori* and obtained meeting dates from publicly available conference announcements. After identifying dates of each individual meeting, we created a composite “meeting” variable that consisted of all surgery conference periods combined. The control group consisted of patients admitted three weeks before and after each meeting.

### Risk adjustment

When modeling the influence of conference periods on mortality, we adjusted the probability of death for patients using the ISS, the motor component of the Glasgow Coma Scale, age, gender, race, initial systolic blood pressure in the Emergency Department, mechanism of injury, inter-hospital transfer status, payer type, and hospital teaching status.

### Analysis

We first performed descriptive statistics using chi-square and Student t tests to compare patient characteristics in both cohorts. All tests were two tailed with α = 0.05. We then performed risk adjustment of patient level factors and hospital teaching status using multivariable logistic regression models, clustering at the hospital level. We constructed the mortality model using forward selection, and the order of variable entry was determined by the c-index, a measure of the ability of a parameter to discriminate outcome. Continuous data (systolic blood pressure) exhibited a right-skewed distribution and was natural log-transformed in a manner consistent with the TQIP mortality model.[14] We tested the influence of each individual meeting on mortality, and we tested the cumulative meeting influence with the composite “meeting” variable. To further examine the relationship between exposure to surgery conference periods and mortality, we stratified patients by injury characteristics (blunt, hypotensive blunt, and penetrating), and we stratified hospitals by ACS and state trauma verification level. Additionally, we stratified the overall study cohort by injury severity (ISS ≤ and ISS > 15) to evaluate the contribution of patient acuity to mortality risk during surgery conferences. In order to assess the influence of the number of trauma surgeons at a given hospital on risk during surgery meetings, we tested for interaction effects between those variables–number of trauma surgeons and meetings–in the overall cohort as well as in hospitals stratified by trauma verification level. We also tested for interaction between meetings and patient characteristics (race, gender, insurance status, and age) to identify an particularly vulnerable subpopulation. Finally, we used posterior prediction models to determine probability of risk-adjusted mortality for each cohort.

### Sensitivity analyses

We performed several sensitivity analyses. We first conducted falsification analyses to assess for potential confounding in the relationship between surgery meeting and non-meeting periods.[15–17] Specifically, we tested for differences in in-patient trauma mortality between meetings and non-meeting dates for oncology (American Society of Clinical Oncology and American Association for Cancer Research) and health services research (Academy Health and American Public Health Association). We then used alternative definitions of our control group (two weeks and four weeks before and after meeting dates rather than three weeks).

## Results

### Patient and hospital characteristics

The ACS TQIP registry included a total of 94,566 trauma patient admissions reported from 156 hospitals during the study period. Of those patients, 12,256 patients (13%) presented during national surgical conferences. Patients in both cohorts, those admitted during meeting compared with non-meeting periods, were similar with respect to their demographic and injury characteristics. The majority of patients were male and white, and falls were the most common mechanism of injury. The majority of patients in each cohort were treated at ACS level I university hospitals, and approximately one third of the patients in each cohort were transferred from other facilities, suggesting that patients were not diverted away from academic centers during surgical conferences. Patient and injury characteristics of each cohort are summarized in Table 1.

**Table 1:** Patient characteristics (n = 94,655).

	Non-conference Admissions (N = 82,399)	Conference Admissions (N = 12,256)	P-value*
Age (years), %			0.50
16–25	17.4	17.5	
26–35	12.7	12.8	
36–55	26.3	26.2	
56–65	13.3	12.8	
66–75	10.1	10.2	
76–85	12.2	12.4	
> 85	5.6	6.0	
Not Recorded/Unknown	2.3	2.2	
Female (%)	35.8	35.6	0.77
Race (%)			0.07
White	72.2	73.0	
African American	13.5	13.1	
Asian	1.7	1.5	
Other	9.5	9.0	
Not Recorded/Unknown	3.2	3.4	
Initial Systolic Blood pressure in Emergency Dept., mean (SD)	138 (30)	139 (30)	0.11
Motor component of the Glasgow Coma Scale (%)			0.10
1	8.4	8.3	
2	0.4	0.3	
3	0.5	0.5	
4	1.7	1.4	
5	3.3	3.6	
6	82.8	83.1	
Not Recorded/Applicable	3.0	3.0	
Patient injury severity using ISS-98, mean (SD)	16.7 (9.0)	16.7 (8.8)	0.97
Mechanism of Injury (%)			0.53
Pedestrian struck	6.3	6.1	
Motor vehicle crash	24.8	24.6	
Cut/pierce	2.9	2.6	
Fall	42.1	42.5	
Firearm	4.8	4.9	
Motorcyclist	6.3	6.5	
Pedestrian other	0.4	0.4	
Other	12.6	12.5	
Transferred from other facility (%)	31.7	31.2	0.50
Teaching status (%)			0.06
Community	33.2	34.3	
Non-teaching	6.4	6.2	
University	60.4	59.6	
American College of Surgeons trauma verification level (%)			
I	55.7	55.5	0.11
II	19.5	20.3	
Not applicable	24.8	24.3	
State trauma verification level			0.43
I	65.2	64.5	
II	16.7	17.2	
Not applicable	17.8	18.0	
Payer status			0.64
Private/commercial	25.4	24.8	
Medicaid	9.1	9.1	
Medicare	26.5	26.6	
Other	33.6	34.0	
Not known/not recorded	5.4	5.5	

* Chi-square used to calculate p-value for categorical variables, and Student t-test used to calculate p-value for continuous variables

The study included data collected from 145 hospitals (2010–2011), with an addition ten hospitals included in 2011. The majority of participating hospitals had an ACS trauma verification level (I or II), however, a considerable number of hospitals without ACS trauma verification contributed data as well (41 hospitals in 2010 and 42 hospitals in 2011). We provided a summary of hospital characteristics in Table 2 and a summary of patients admitted to state-verified level I trauma centers that lack ACS verification in Appendix A in S1 Table.

**Table 2:** Hospital characteristics by state and American College of Surgeons (ACS) trauma verification levels (n = 155).

	State level		
	I (n = 95)	II (n = 33)	NA (n = 27)
ACS level I, n (%)	60 (77.9)	0	17 (22.1)
Number of beds (%)			
< = 200	0	—	0
201–400	8 (13.3)	—	3 (17.7)
401–600	14 (23.3)	—	6 (35.3)
>600	38 (63.3)	—	8 (47.1)
Teaching status, n (%)			
Community	14 (23.3)	—	4 (23.5)
Non-teaching	1 (1.7)	—	0
University	45 (75)	—	13 (76.5)
Number of trauma surgeons, median (IQR)	7 (6–8)	—	6 (5–7)
ACS level II, n(%)	3 (7.1)	29 (69.1)	9 (21.4)
Number of beds, n (%)			
< = 200	0	3 (10.3)	0
201–400	3 (100)	12 (41.4)	5 (55.6)
401–600	0	8 (27.6)	4 (44.4)
>600	0	6 (20.7)	0
Teaching status, n (%)			
Community	2 (66.7)	20 (69.0)	6 (66.7)
Non-teaching	1 (33.3)	4 (13.8)	1 (11.1)
University	0	5 (17.2)	2 (22.2)
Number of trauma surgeons, median (IQR)	4 (4–6)	6 (5–7)	4 (4–5)
ACS level–NA, n (%)	32 (86.5)	4 (10.8)	1 (2.7)
Number of beds (%)			
< = 200	0	0	0
201–400	2 (6.3)	0	0
401–600	13 (40.6)	2 (50.0)	1 (100)
>600	17 (53.1)	2 (50.0)	0
Teaching status (%)			
Community	12 (37.5)	3 (75.0)	0
Non-teaching	3 (9.4)	1 (25.0)	0
University	17 (53.1)	0	1 (100)
Number of trauma surgeons, median (IQR)	6 (5–9)	6 (4–8)	5 (NA)

### Mortality during meeting and non-meeting periods

Overall risk-adjusted in-patient mortality was similar for trauma patients admitted during meetings compared with non-meeting periods (approximately 6%). Also, when we stratified patients by injury type (blunt and penetrating), patient mortality exhibited no difference between meeting and non-meeting cohorts. However, we found that when stratified by ACS trauma verification level, mortality increased significantly during meetings among trauma patients admitted to hospitals that lacked ACS trauma verification (OR 1.2, p = 0.008). That association was particularly pronounced at non-ACS verified trauma centers among patients with penetrating injuries, whose predicted mortality increased from 12% to 16%. We found in subgroup analysis by state verification level that the conference effect in non-ACS trauma centers was entirely driven by state verified level I trauma centers. In contrast, mortality remained unchanged during meetings compared with non-meeting periods for injured patients who presented to ACS verified trauma centers.

**Fig 1:**
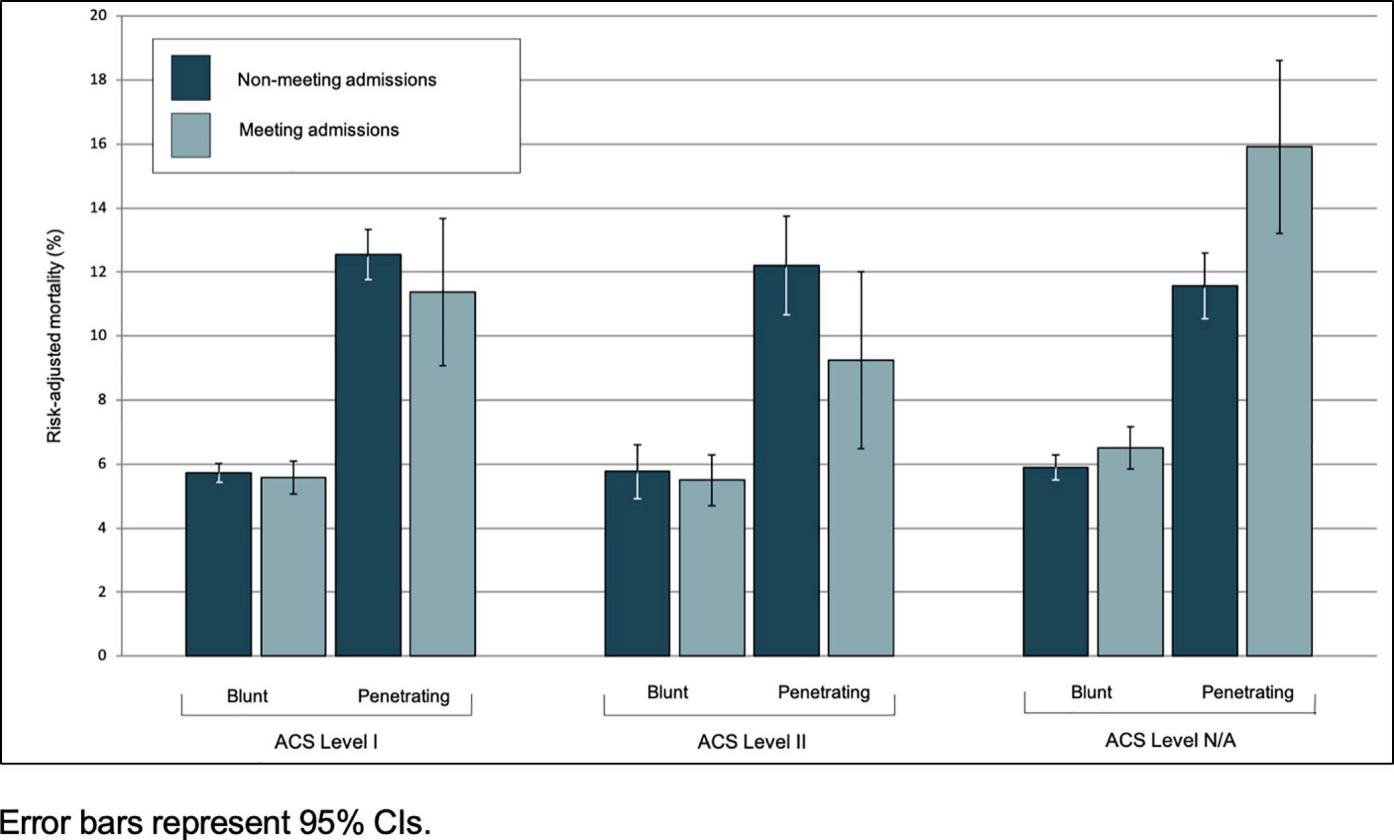
Adjusted mortality during national surgery meeting and non-meeting periods by American College of Surgeons (ACS) trauma verification level and injury characteristics. Error bars represent 95% CIs.

When stratified by injury acuity (ISS≤15 and ISS>15), we found no evidence in the overall patient cohort of conference effect among low-acuity patients (OR 1.1, p = 0.47) and high-acuity patients (OR 0.9, p = 0.74). At trauma centers that lacked ACS trauma verification, we found statistically insignificant trends towards increased mortality during meetings among both low- and high-acuity patients (OR 1.47, p = 0.5 and OR 1.16, p = 0.09, respectively). The association only reached statistical significance among high-acuity patients with penetrating injuries (OR 2.15, p = 0.03). We found no evidence of interaction effects between number of trauma surgeons and meetings, meaning the number of trauma surgeons did not contribute to mortality differences during meetings, so we did not include that variable in the final mortality model. Additionally, we found no evidence of interaction between patient characteristics (age, gender, race, and payer status) and surgery meetings with regards to mortality risk. Odds ratios and predicted probability of risk-adjusted in-patient mortality during meetings compared with non-meeting periods are summarized for each patient cohort in Table 3 and Fig 1, respectively.

**Table 3:** Adjusted mortality during national surgery meetings compared with non-meeting periods by American College of Surgeons (ACS) trauma verification level, injury characteristics, and state trauma verification level for hospitals without ACS trauma verification (n = 94,655).

	Odds ratio	95% CI	P value
Trauma verification level			
Injury characteristic			
Overall	1.0	0.9–1.1	0.88
Blunt	1.0	0.9–1.1	0.89
Blunt hypotensive*	1.2	0.9–1.6	0.36
Penetrating	0.9	0.6–1.3	0.61
ACS level I			
Injury characteristic			
Overall	0.9	0.8–1.1	0.32
Blunt	1.0	0.8–1.1	0.58
Blunt hypotensive*	1.2	0.9–1.8	0.24
Penetrating	0.8	0.5–1.3	0.34
ACS level II			
Injury characteristic			
Overall	0.9	0.7–1.1	0.27
Blunt	0.9	0.7–1.2	0.57
Blunt hypotensive*	0.9	0.4–2.3	0.87
Penetrating	0.5	0.3–1.1	0.10
ACS NA			
Injury characteristic			
Overall	1.2	1.1–1.4	0.008
Blunt	1.2	1.0–1.3	0.06
Blunt hypotensive*	0.9	0.5–1.7	0.84
Penetrating	2.5	1.3–4.9	0.006
State level I, ACS level NA			
Injury characteristic			
Overall	1.3	1.1–1.5	0.001
Blunt	1.2	1.1–1.4	0.008
Blunt hypotensive*	0.8	0.4–1.5	0.53
Penetrating	3.1	1.5–6.1	0.001
State level II, ACS level NA			
Injury characteristic			
Overall	0.8	0.4–1.5	0.48
Blunt	0.8	0.4–1.5	0.45
Blunt hypotensive*	0.1	0.0–2.4	0.17
Penetrating	0.7	0.1–9.2	0.78
State level NA, ACS level NA			
Injury characteristic			
Overall	0.4	0.1–1.0	0.05
Blunt	0.4	0.1–1.0	0.06
Blunt hypotensive*	—	—	—
Penetrating	—	—	—

*Hypotensive = initial systolic blood pressure < 90

“—” Model unable to converge

### Sensitivity analyses

We tested the robustness of our model and found no evidence that unmeasured confounding contributed to differences in in-patient mortality between national surgical meetings and non-meeting periods. Risk-adjusted mortality of trauma patients was similar during national meetings for oncology and health services research when compared with the three weeks before and after those meetings. That analysis included 114,918 patients, 17,154 patients admitted during meetings and 97,764 patients admitted during non-meeting periods. Subgroup analysis, stratifying patients by hospital ACS trauma verification level and injury type, produced no difference in mortality between cohorts (meeting and non-meeting) for non-surgery conferences. Results of those tests are summarized in Table 4. Also, when we defined control groups as patients admitted two weeks and four weeks before and after surgical meetings, we found results similar to our initial analysis; changes in mortality were not associated with surgery meetings for patients admitted to ACS verified trauma centers, but mortality increased significantly for patients admitted to non-verified trauma centers during meetings, particularly if they presented with penetrating injuries. Results of this sensitivity analysis are summarized in Appendix B in S1 Table.

**Table 4:** Adjusted mortality during oncology and health services research meetings compared with non-meeting periods by American College of Surgeons (ACS) trauma verification level and injury characteristics during. Patient N = 114,918.

	Odds ratio	95% CI	P value
All hospitals			
Injury characteristic			
Overall	0.9	0.8–1.0	0.60
Blunt	0.9	0.8–1.0	0.14
Blunt hypotensive*	1.2	0.8–1.6	0.10
Penetrating	0.8	0.6–1.0	0.39
ACS level I			
Injury characteristic			
Overall	0.9	0.8–1.1	0.40
Blunt	1.0	0.8–1.1	0.51
Blunt hypotensive*	1.4	0.9–2.3	0.14
Penetrating	0.9	0.5–1.3	0.47
ACS level II			
Injury characteristic			
Overall	0.9	0.7–1.0	0.14
Blunt	0.9	0.7–1.1	0.24
Blunt hypotensive*	1.2	0.4–3.6	0.69
Penetrating	0.6	0.2–1.9	0.43
Not Applicable			
Injury characteristic			
Overall	0.9	0.7–1.1	0.21
Blunt	0.9	0.7–1.1	0.37
Blunt hypotensive*	0.6	0.3–1.1	0.10
Penetrating	0.7	0.4–1.1	0.15

*Hypotensive = initial systolic blood pressure < 90

## Discussion

We found substantial increases in mortality among trauma patients admitted to hospitals that lack trauma center verification by the ACS during national surgery conferences, particularly for patients who presented with penetrating injuries. Since typical risk factors such as ISS, age, and transfer status did not differ significantly between patients admitted during meeting and non-meeting periods, it is unlikely that high-risk patients were directed to or from particular hospitals during meetings. The increased mortality during meetings is most likely attributable to the selective decline of surgeons and changes in hospital staff composition that occur, potentially leaving hospitals with decreased capacity and/or capability to effectively treat injured patients. The finding that mortality increased among patients with penetrating injuries, in particular, supports this explanation, since those patients often require surgical intervention compared with patients whose injuries result from blunt mechanisms.

Study of the conference effect phenomenon is limited. Cardiology conferences have been associated with decreases in both percutaneous coronary interventions (PCI) and cardiovascular mortality, whereas we found surgery conferences were associated with increased mortality for trauma patients.[11] These differing results are likely attributable to differences in the therapeutic options and timing of death for each patient population. While evidence exists that patients with cardiovascular disease may tolerate non-invasive medical management or delayed PCI, the timing of treatment of traumatic injuries, particularly penetrating injuries, is less ambiguous.[18, 19] Results of this study, however, are consistent with the findings of other studies of predictable fluctuations in staffing such as the “weekend effect”[1, 2] and the “July effect”[3–5] and may prompt hospitals with limited surgical capacity to critically examine staffing during national surgery meetings. Organizers of those conferences may also consider providing additional opportunities for teleconferencing so that at-risk trauma centers can maintain their capacity, while still enabling their surgeons to benefit from the educational opportunities the conferences afford.

It is also noteworthy that we found no such association between surgery meetings and increased mortality for patients admitted to ACS verified trauma centers. This finding may be due to trauma center adherence to the ACS guidelines. The American College of Surgeons Committee on Trauma (ACS-COT) has maintained guidelines for regionalized trauma systems since 1979,[20] and the ACS-COT implemented trauma center verification processes for trauma centers in 1987 that outline necessary resources and personnel to treat the injured patient.[21] Prior studies suggest that ACS-COT guidelines have been associated with substantial reductions in trauma mortality.[22–24] Our findings support the existing body of evidence that requirements of trauma center verification protect against clinically significant fluctuations in hospital staffing.[1, 6–8, 10] Of note, the baseline number of trauma surgeons at an institution did not contribute to the influence of meetings on mortality. Potential explanations for this finding include that the baseline number of surgeons at a hospital reflect neither meeting attendance nor the composition of surgeons who remain at hospitals during meetings with regards to their clinical experience.

The primary limitation of our study was the inability to establish specific mechanisms by which patients admitted during national surgery conferences to hospitals lacking ACS verification had increased risk of mortality. Further study of variation in processes of care such as resuscitation practices and procedures may explain the influence of surgical meetings on clinical outcomes. Another limitation is that we selected four surgery conferences *a priori* for the purposes of this study. The study does not include a comprehensive assessment of all surgery, trauma, and critical care conferences. Inclusion of both additional specialty conferences and more granular attendance data, including the clinical experience of the attendees, may clarify the relationship between fluctuations in staffing during surgery conferences and trauma patient mortality.

### Conclusions

In this study, we identified a “conference effect” for trauma patients treated at non-ACS verified trauma centers, and we identified patients with penetrating injuries as particularly at-risk of increased mortality during national surgery meetings. Staffing criteria of trauma center verification by the American College of Surgeons appears to protect against the conference effect. These findings have important implications for both how hospitals prepare for conferences with regards to staffing and, potentially, how conferences themselves are structured to allow for increased remote participation.

## Supporting information

S1 Table Appendix A and Appendix B.
(DOCX)

S1 File (DTA)
