# Supplementary material for: The conference effect: National surgery meetings are associated with increased mortality at trauma centers without American College of Surgeons verification
Source: PLoS One. 2019 Mar 26;14(3):e0214020. doi: 10.1371/journal.pone.0214020 (PMC6435237; doi:10.1371/journal.pone.0214020)
Supplement: S1 Table — Appendix A and Appendix B. (DOCX) [file pone.0214020.s001.docx]

Appendix A. Patient characteristics at state verified level I trauma centers without ACS trauma verification (n=18,766)

|  | Non-conference Admissions (N=16,384) | Conference Admissions  (N=2,382) | P-value* |
| --- | --- | --- | --- |
| Age (years), % |  |  | 0.26 |
| 16-25 | 17.3 | 17.7 |  |
| 26-35 | 12.5 | 13.4 |  |
| 36-55 | 26.5 | 25.9 |  |
| 56-65 | 13.1 | 11.6 |  |
| 66-75 | 9.9 | 10.2 |  |
| 76-85 | 12.5 | 12.9 |  |
| > 85 | 6.9 | 6.3 |  |
| Not Recorded/Unknown | 2.4 | 1.9 |  |
| Female (%) | 36.2 | 36.8 | 0.80 |
| Race (%) |  |  | 0.15 |
| White | 73.6 | 74.4 |  |
| African American | 19.5 | 19.1 |  |
| Asian | 1.0 | 0.7 |  |
| Other | 4.0 | 3.3 |  |
| Unknown | 2.0 | 2.5 |  |
| Initial Systolic Blood pressure in Emergency Dept., mean (SD) | 139.6 | 139.7 | 0.52 |
| Motor component of the Glasgow Coma Scale (%) |  |  | 0.71 |
| 1 | 8.2 | 8.5 |  |
| 2 | 0.4 | 0.3 |  |
| 3 | 0.5 | 0.3 |  |
| 4 | 1.7 | 1.3 |  |
| 5 | 3.6 | 3.8 |  |
| 6 | 83.5 | 83.7 |  |
| Not Recorded/Applicable | 2.3 | 2.0 |  |
| Patient injury severity using ISS-98, mean (SD) | 17.0 | 16.9 | 0.44 |
| Mechanism of Injury (%) |  |  | 0.59 |
| Pedestrian struck | 5.6 | 4.7 |  |
| Motor vehicle crash | 26.7 | 27.4 |  |
| Cut/pierce | 2.8 | 2.9 |  |
| Fall | 42.6 | 42.9 |  |
| Firearm | 5.4 | 5.3 |  |
| Motorcyclist | 5.7 | 6.1 |  |
| Pedestrian other | 0.3 | 0.2 |  |
| Other | 10.9 | 10.6 |  |
| Transferred from other facility (%) | 29.6 | 28.9 | 0.48 |
| Teaching status (%) |  |  | 0.02 |
| Community | 29.3 | 30.1 |  |
| Non-teaching | 5.7 | 4.2 |  |
| University | 65.1 | 65.7 |  |
| Payer status |  |  | 0.50 |
| Private/commercial | 23.5 | 22.8 |  |
| Medicaid | 8.3 | 8.7 |  |
| Medicare | 25.0 | 26.4 |  |
| Other | 32.6 | 32.2 |  |
| Not known/not recorded | 10.6 | 10.0 |  |

* Chi-square used to calculate p-value for categorical variables, and Student t-test used to calculate p-value for continuous variables

Appendix B. Adjusted mortality during national surgery meetings compared with non-meeting periods by American College of Surgeons (ACS) trauma verification level and injury characteristics with non-meeting periods set at two weeks (n=73,777) and four weeks (n=110,113) before and after surgery conferences

|  | Odds ratio | 95% CI | P value |
| --- | --- | --- | --- |
| **Two Weeks** |  |  |  |
| All hospitals |  |  |  |
| Injury characteristic |  |  |  |
| Overall | 1.0 | 0.9-1.1 | 0.47 |
| Blunt | 1.0 | 0.9-1.1 | 0.59 |
| Blunt hypotensive* | 1.1 | 0.8-1.5 | 0.41 |
| Penetrating | 0.9 | 0.7-1.4 | 0.74 |
| ACS level I |  |  |  |
| Injury characteristic |  |  |  |
| Overall | 0.9 | 0.8-1.0 | 0.10 |
| Blunt | 0.9 | 0.8-1.1 | 0.23 |
| Blunt hypotensive* | 1.2 | 0.8-1.7 | 0.29 |
| Penetrating | 0.7 | 0.4-1.3 | 0.38 |
| ACS level II |  |  |  |
| Injury characteristic |  |  |  |
| Overall | 0.9 | 0.7-1.1 | 0.21 |
| Blunt | 0.9 | 0.7-1.1 | 0.41 |
| Blunt hypotensive* | 0.8 | 0.3-2.1 | 0.67 |
| Penetrating | 0.6 | 0.3-1.3 | 0.19 |
| Not Applicable |  |  |  |
| Injury characteristic |  |  |  |
| Overall | 1.2 | 1.1-1.4 | 0.007 |
| Blunt | 1.2 | 1.0-1.4 | 0.09 |
| Blunt hypotensive* | 1.1 | 0.6-1.9 | 0.81 |
| Penetrating | 2.6 | 1.3-5.0 | 0.005 |
| **Four Weeks** |  |  |  |
| All hospitals |  |  |  |
| Injury characteristic |  |  |  |
| Overall | 1.0 | 0.9-1.1 | 0.92 |
| Blunt | 1.0 | 0.9-1.1 | 0.74 |
| Blunt hypotensive* | 1.2 | 0.9-1.6 | 0.33 |
| Penetrating | 0.9 | 0.7-1.3 | 0.67 |
| ACS level I |  |  |  |
| Injury characteristic |  |  |  |
| Overall | 1.0 | 0.8-1.1 | 0.42 |
| Blunt | 1.0 | 0.8-1.1 | 0.62 |
| Blunt hypotensive* | 1.2 | 0.8-1.8 | 0.30 |
| Penetrating | 0.8 | 0.5-1.3 | 0.45 |
| ACS level II |  |  |  |
| Injury characteristic |  |  |  |
| Overall | 0.9 | 0.7-1.1 | 0.34 |
| Blunt | 0.9 | 0.8-1.2 | 0.65 |
| Blunt hypotensive* | 1.0 | 0.4-2.4 | 0.97 |
| Penetrating | 0.6 | 0.3-1.2 | 0.14 |
| Not Applicable |  |  |  |
| Injury characteristic |  |  |  |
| Overall | 1.2 | 1.1-1.4 | 0.008 |
| Blunt | 1.2 | 1.0-1.4 | 0.04 |
| Blunt hypotensive* | 1.0 | 0.6-1.8 | 0.97 |
| Penetrating | 2.0 | 1.1-3.6 | 0.02 |

*Hypotensive = initial systolic blood pressure < 90
